# A case report: Becker muscular dystrophy presenting with epilepsy and dysgnosia induced by duplication mutation of Dystrophin gene

**DOI:** 10.1186/s12883-016-0777-y

**Published:** 2016-12-12

**Authors:** Jing Miao, Jia-chun Feng, Dan Zhu, Xue-fan Yu

**Affiliations:** Department of Neurology and Neuroscience Center, The First Affiliated Hospital of Jilin University, Changchun, 130021 Jilin People’s Republic of China

**Keywords:** Becker muscular dystrophy, Epilepsy, Dysgnosia, Dystrophin, Genetic analysis

## Abstract

**Background:**

Becker muscular dystrophy (BMD), a genetic disorder of X-linked recessive inheritance, typically presents with gradually progressive muscle weakness. The condition is caused by mutations of Dystrophin gene located at Xp21.2. Epilepsy is an infrequent manifestation of BMD, while cases of BMD with dysgnosia are extremely rare.

**Case presentation:**

We describe a 9-year-old boy with BMD, who presented with epilepsy and dysgnosia. Serum creatine kinase level was markedly elevated (3665 U/L). Wechsler intelligence tests showed a low intelligence quotient (IQ = 65). Electromyogram showed slight myogenic changes and skeletal muscle biopsy revealed muscular dystrophy. Immunohistochemical staining showed partial positivity of sarcolemma for dystrophin-N. Multiplex ligation-dependent probe amplification revealed a duplication mutation in exons 37–44 in the Dystrophin gene.

**Conclusions:**

The present case report helps to better understand the clinical and genetic features of BMD.

## Background

Becker muscular dystrophy (BMD), a genetic disorder of X-linked recessive inheritance, is caused by mutations of Dystrophin gene located at Xp21.2 [1]. The disease typically manifests as progressive muscle weakness of the extremities and pelvic muscles. However, an estimated 7.54% BMD cases may present as epilepsy [2]. Cases of BMD with concomitant dysgnosia are extremely rare [3–5]. Herein, we report a case of BMD which presented with epilepsy and dysgnosia as the initial symptom.

## Case presentation

A 9-year-old boy presented to us with a 2-year-long history of episodic epileptic seizures. His growth and development milestones were delayed compared to his peers. He started walking at the age of 14 months and had poor speech and mathematical ability. However, the boy was able to walk and run normally. At the age of seven years, he experienced his first epileptic seizure which lasted for about 1 minute. The seizure manifested as convulsions with sudden loss of consciousness, turning of eyes and head tilt to the left side, and limb rigidity (left-arm extension; right-arm flexion mimicking an archery posture; lower-extremity flexion). There was no history of odaxesmus or urinary incontinence. Seizure episodes recurred at an irregular frequency. His past medical history and family history were unremarkable.

On examination, he showed poor ability for mathematical calculations (93–7 = ?), dysarthria and bilateral enlargement of gastrocnemius muscles. Muscle strength in the proximal muscles was 5-/5. No cranial nerve dysfunction, sensory disturbances, or ataxia was noted. All nerve reflexes were normal.

**Fig. 1:**
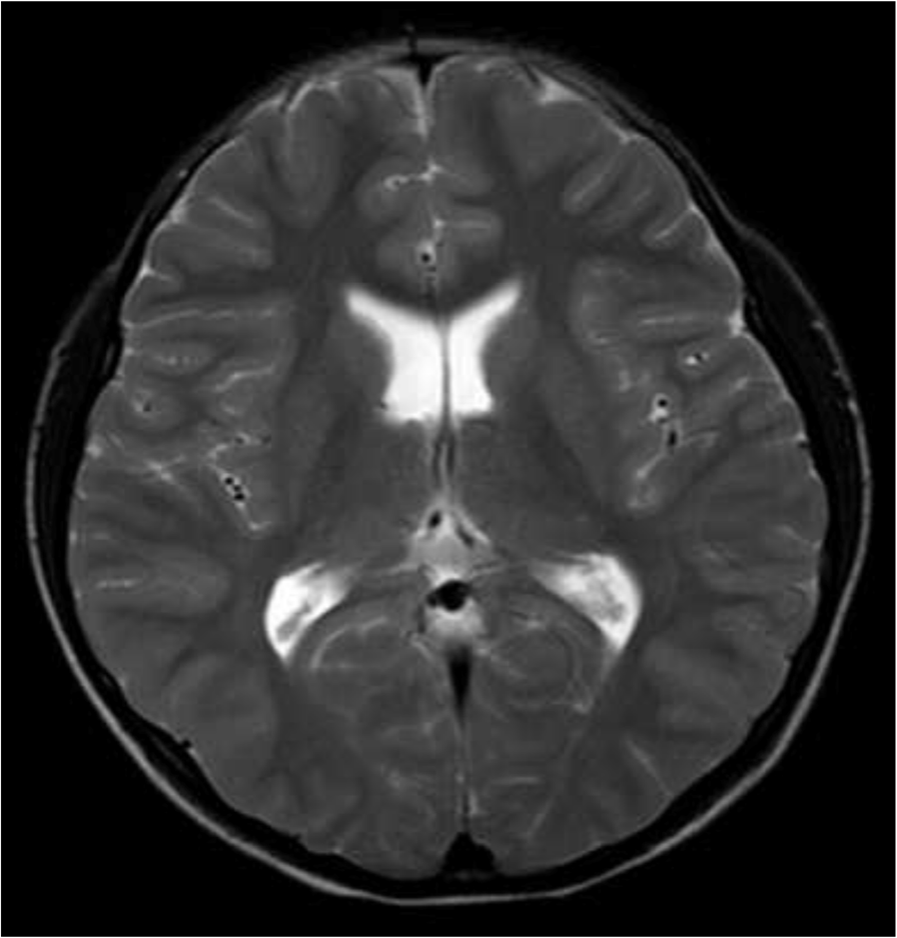
Cranial magnetic resonance imaging (MRI) was normal

Laboratory examination at one week after onset showed an elevated serum creatine kinase level (3665 U/L; normal range 25–200 U/L). Cranial magnetic resonance imaging (MRI) was normal (Fig. 1). Wechsler intelligence tests revealed a low intelligence quotient (IQ): total IQ 65 (normal range, 90–109); manual IQ 63; and language IQ 72. Electromyogram showed slight myogenic changes.

Electroencephalography showed abundant interparoxysmal multifocal spike-slow complex waves and sharp-slow complex waves in bilateral central-parietal areas and frontal midline area, which is consistent with partial seizures. Skeletal muscle biopsy (biceps muscle) was performed after obtaining written consent of his parents. After precooling with isopentane, the muscle specimens were frozen in liquid nitrogen. Frozen sections (8 μm) were prepared and histopathological and immunohistochemical staining performed using hematoxylin-eosin (HE), modified Gomori trichrome (MGT), NADH-tetrazolium reductase (NADH-TR), succinate dehydrogenase (SDH), cytochrome c oxidase (COX), acid phosphatase, periodic acid-schiff (PAS), oil red O, dystrophin-N/C/R, sarcoglycan-α/β/γ/δ, dysferlin, and major histocompatibility complex 1 (MHC-1).

**Fig. 2:**
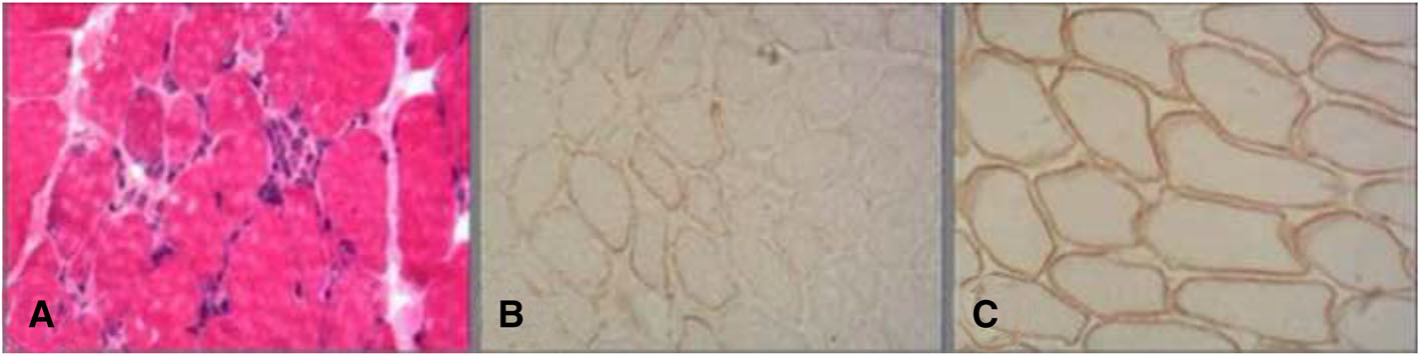
Histopathological examination (**a**, hematoxylin-eosin staining, ×200) showing muscle fibers of variable sizes, few degenerative, necrotic and regenerative muscle fibers, and moderate connective tissue hyperplasia. Immunohistochemical staining showed partial positivity of sarcolemma for dystrophin-N (**b**, ×200) as compared to normal control (**c**, ×200)

Histopathological examination showed a few degenerative, necrotic and regenerative muscle fibers and moderate connective tissue hyperplasia; staining for glycogen and lipids was negative. All enzymatic examinations were normal. On immunohistochemical staining, sarcolemma showed partly positivity for dystrophin-N when compared with normal control (Fig. 2).

**Fig. 3:**
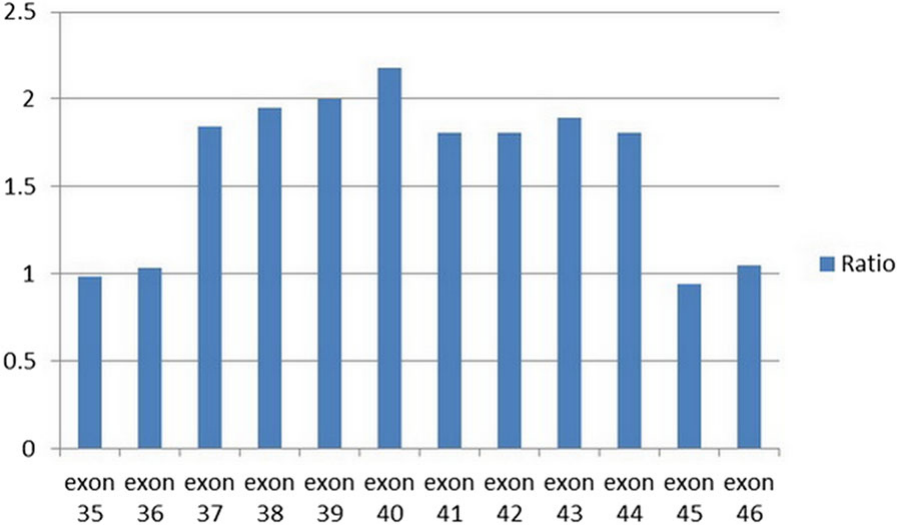
Multiplex ligation-dependent probe amplification of the DNA extracted from peripheral blood showed a duplication mutation in exons 37–44 in the Dystrophin gene (fluorescence intensity 1.81–2.18; normal range 0.7–1.33)

Multiplex ligation-dependent probe amplification (MLPA) showed a duplication mutation in exons 37–44 in the Dystrophin gene. Additionally, we searched the gene bank (HGMD Pro, PubMed, 1000Genomes, and dbSNP databases), and confirmed that this mutation was novel. Genetic testing of his parents revealed that this gene variant was inherited from his mother (Fig. 3). Based on the clinical and genetic evidence, a definitive diagnosis of BMD was made. In addition to the prescribed [0.3 g (a.m.) and 0.6 g (p.m.)] twice daily oxcarbazepine, the patient was administered 10 mg per day coenzyme Q10 for approximately 2 years. Two years later his symptoms relieved and an EEG showed less spikes and slow waves than it had previous shown. He had no adverse events and his motor function remained stable.

## Conclusions

The incidence of BMD is estimated to be 1.5 to 6 per 100,000 male births. Symptoms classically appear between the ages of 8–25 years [6]. The diagnostic tests include muscle biopsy, creatine kinase test, electromyography and genetic testing.

The current case manifested delayed growth and development, and physical examinations showed proximal weakness in all extremities. Serum creatine kinase level was elevated. Electromyogram showed slight myogenic changes and immunohistochemical staining revealed skeletal sarcolemma was partly positive for dystrophin-N. DNA sequencing analysis showed a duplication mutation in exons 37–44 in the Dystrophin gene. He met all the above diagnostic criteria of BMD.

Cases of BMD presenting with epilepsy and dysgnosia as initial symptoms are rare in the published literature. However, the muscular involvement in this boy was mild. Cases of BMD patients with mental disorders such as autism and inattention are also on record [7].

Immunohistochemical staining showed that the skeletal sarcolemma was partly positive for dystrophin-N, which suggests that the N-terminal of dystrophin protein was significantly involved. Dystrophin protein is a rod-shaped cytoplasmic protein located at the sarcolemmal surface. It comprises of three domains: an N-terminal actin-binding domain, an R-terminal domain in the middle stalk region, and a unique C-terminal domain involving a vital part of the dystrophin-associated glycoprotein complex (DGC) that links the cytoskeleton protein to the ion channels and the signal proteins at the surface of the cytoplasmic membrane [8]. The mild muscular involvement in this case may be associated with mild impairment of DGC functions, caused by partial deficiency of the N-terminal of dystrophin protein.

Dystrophin is encoded by one of the largest and most complex human genes, which comprise of 79 exons. Dystrophin gene encodes for eight isoproteins and seven of these are expressed in the central nervous system: 3 full-length dystrophin (3 variants of Dp427), and 4 shorter non-muscle products (Dp260, Dp140, Dp116, and Dp71) [9]. The abnormalities of Dystrophin gene may result in structural and functional deficiency of dystrophin protein, which affects the contraction and relaxation functions of muscles.

The duplication mutation in exons 37–44 in the Dystrophin gene detected in this case has not been reported previously. We speculate that this mutation affects the structure and function of Dp140, and is likely to be a pathogenetic variant [10]. Deficiency of Dp140 has been shown to result in epilepsy by increasing neuronal excitability (by reducing the receptors of inhibitory neurotransmitter GABA_A_ and by affecting the ion channels on the surface membranes of cells) [11]. Dp140 can also induce dysgnosia by reducing the volume of gray matter and by affecting the information processing ability [3].

Considering the main clinical manifestations of epilepsy, dysgnosia, and muscular dystrophy, the differential diagnoses should include Fukuyama congenital muscular dystrophy (FCMD), limb-girdle muscular dystrophy [12–14]. These conditions may present with similar symptoms. The definitive diagnosis depends on muscle biopsy and genetic analysis. In the future, more and more cases will be required to more completely understand and confirm genotype-phenotype relationships.

## Abbreviations

BMD: Becker muscular dystrophy
MRI: Magnetic resonance imaging
IQ: Intelligence quotient
HE: Hematoxylin-eosin
MGT: Modified Gomori trichrome, NADH-TR, NADH-tetrazolium reductase
SDH: Succinate dehydrogenase
COX: Cytochrome c oxidase
PAS: Periodic acid-schiff
MHC-1: Major histocompatibility complex 1
MLPA: Multiplex ligation-dependent probe amplification
DGC: Dystrophin-associated glycoprotein complex
FCMD: Fukuyama congenital muscular dystrophy
